# A mosquito small RNA genomics resource reveals dynamic evolution and host responses to viruses and transposons

**DOI:** 10.1101/gr.265157.120

**Published:** 2021-03

**Authors:** Qicheng Ma, Satyam P. Srivastav, Stephanie Gamez, Gargi Dayama, Fabiana Feitosa-Suntheimer, Edward I. Patterson, Rebecca M. Johnson, Erik M. Matson, Alexander S. Gold, Douglas E. Brackney, John H. Connor, Tonya M. Colpitts, Grant L. Hughes, Jason L. Rasgon, Tony Nolan, Omar S. Akbari, Nelson C. Lau

**Affiliations:** 1 Department of Biochemistry, Boston University School of Medicine, Boston, Massachusetts 02118, USA;; 2 Division of Biological Sciences, Section of Cell and Developmental Biology, University of California San Diego, La Jolla, California 92093, USA;; 3 Department of Microbiology and the National Emerging Infectious Disease Laboratory, Boston University School of Medicine, Boston, Massachusetts 02118, USA;; 4 Departments of Vector Biology and Tropical Disease Biology, Centre for Neglected Tropical Diseases, Liverpool School of Tropical Medicine, Liverpool L3 5QA, United Kingdom;; 5 Department of Entomology, Center for Infectious Disease Dynamics, and the Huck Institutes for the Life Sciences, Pennsylvania State University, University Park, Pennsylvania 16802, USA;; 6 Department of Environmental Sciences, The Connecticut Agricultural Experiment Station, New Haven, Connecticut 06511, USA;; 7 Boston University Genome Science Institute and the National Emerging Infectious Disease Laboratory, Boston, Massachusetts 02118, USA

## Abstract

Although mosquitoes are major transmission vectors for pathogenic arboviruses, viral infection has little impact on mosquito health. This immunity is caused in part by mosquito RNA interference (RNAi) pathways that generate antiviral small interfering RNAs (siRNAs) and Piwi-interacting RNAs (piRNAs). RNAi also maintains genome integrity by potently repressing mosquito transposon activity in the germline and soma. However, viral and transposon small RNA regulatory pathways have not been systematically examined together in mosquitoes. Therefore, we developed an integrated mosquito small RNA genomics (MSRG) resource that analyzes the transposon and virus small RNA profiles in mosquito cell cultures and somatic and gonadal tissues across four medically important mosquito species. Our resource captures both somatic and gonadal small RNA expression profiles within mosquito cell cultures, and we report the evolutionary dynamics of a novel Mosquito-Conserved piRNA Cluster Locus (MCpiRCL) made up of satellite DNA repeats. In the larger culicine mosquito genomes we detected highly regular periodicity in piRNA biogenesis patterns coinciding with the expansion of Piwi pathway genes. Finally, our resource enables detection of cross talk between piRNA and siRNA populations in mosquito cells during a response to virus infection. The MSRG resource will aid efforts to dissect and combat the capacity of mosquitoes to tolerate and spread arboviruses.

Mosquitoes are one of the most prevalent vectors of human pathogens, yet they have wide variability to vector different pathogens. For example, human malaria parasites are exclusively vectored by anopheline mosquitoes, which transmit few viruses other than O'Nyong nyong virus (ONNV) and Mayaro virus (Vanlandingham et al. 2006; Brustolin et al. 2018). In contrast, culicine mosquitoes transmit many human viral pathogens, such as dengue virus (DENV), Zika virus (ZIKV), Chikungunya virus (CHIKV), and yellow fever virus (YFV) in tropical climates where *Aedes albopictus* (*AeAlbo*) and *Aedes aegypti* (*AeAeg*) thrive; and eastern equine encephalitis virus (EEEV) and West Nile Virus (WNV) spread mainly in *Culex* mosquitoes inhabiting temperate climates (Olson and Blair 2015; Londono-Renteria and Colpitts 2016; Halbach et al. 2017; Lambrechts and Saleh 2019).

Because vector-pathogen interactions are complex, no dominant theory yet explains why anopheline mosquitoes are less prolific than culicine mosquitoes in spreading arboviruses. Arbovirus infections in humans lead to devastating symptoms including fever, nausea, bleeding, extreme pain, brain damage, and death. However, culicine mosquitoes are practically unaffected from active arbovirus replication (Goic and Saleh 2012; Olson and Blair 2015; Lambrechts and Saleh 2019) and therefore are highly competent transmitters of arboviruses to human hosts.

Three main classes of animal small regulatory RNAs are microRNAs (miRNAs) and endogenous small interfering RNAs (endo-siRNAs), which range in size between 18 and 23 nt long and are typically bound by Argonaute proteins; and Piwi-interacting RNAs (piRNAs) that are bound by Piwi proteins and mainly range in size between 24 and 32 nt in length in most animals. In the model Dipteran, *Drosophila melanogaster* (*Dmel*), the small RNAs comprise 258 miRNA genes (Kozomara et al. 2019), approximately 20 large intergenic piRNA cluster loci (Brennecke et al. 2007; Malone et al. 2009; Wen et al. 2014), more than 1000 genic piRNA cluster loci (Robine et al. 2009; Wen et al. 2014; Chirn et al. 2015), and more than 1000 endogenous siRNA loci generating either large fold-back transcripts or sense–antisense pairing transcripts (Czech et al. 2008; Ghildiyal et al. 2008; Kawamura et al. 2008; Mirkovic-Hösle and Förstemann 2014; Wen et al. 2014, 2015). Last, arbovirus-specific siRNAs and piRNAs persist in *Dmel* cell cultures (Flynt et al. 2009; Wu et al. 2010; Vodovar et al. 2011; Goic et al. 2013; Wen et al. 2014; Palmer et al. 2018).

Culicidae mosquitoes are relatives of Drosophilid fruit flies as members of the Dipteran insect clade (Fig. 1A; Wiegmann et al. 2011), yet ∼260 million years (MY) of evolutionary distance between Drosophilids and Culicidae imparts physiological and molecular differences in small RNA compositions. Within mosquito phylogeny, the anopheline subclade represented by *Anopheles gambiae* (*AnGam*) displays stronger chromosome synteny to Drosophilids than the culicine subclade of mosquitoes, such as *Culex quinquefasciatus* (*CuQuin*), *Aedes aegypti* (*AeAeg*), and *Aedes albopictus* (*AeAlbo*) (Dudchenko et al. 2017). Indeed, *AnGam*’s genome (∼0.28 Gb) is as compact as *Dmel*’s genome (∼0.18 Gb), whereas culicine mosquito genomes are an order of magnitude greater in size owing to numerous noncoding and repetitive elements (Fig. 1C; Rai and Black 1999; Holt et al. 2002; Nene et al. 2007; Arensburger et al. 2010; Chen et al. 2015; Dudchenko et al. 2017; Matthews et al. 2018; Palatini et al. 2020).

**Figure 1.**
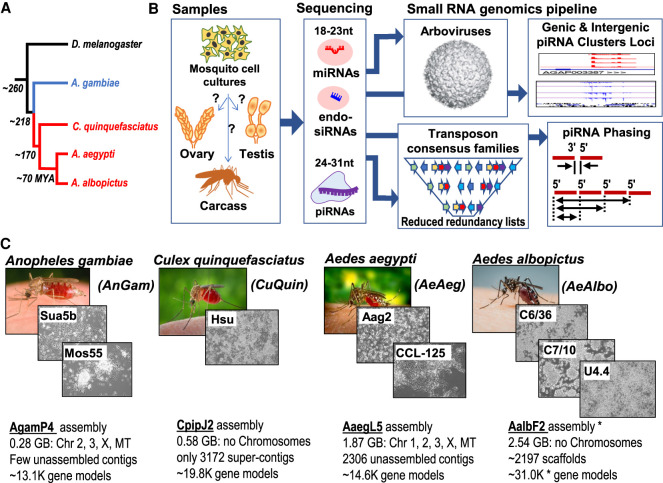
Overview of the mosquito small RNA genomics resource. (*A*) Phylogenetic tree of Dipteran insects in this study, with evolutionary distance measured by million years ago (MYA). Blue and red color denote the anopheline and culicine lineages. (*B*) Organization of this resource that compares mosquito cell cultures to tissue types via determining the small RNA types and their genomic profiles. (*C*) Overview of the four mosquito species genomes and eight cell culture lines subjected to the small RNA genomics analysis pipeline. The specific genome assembly names are noted with genome configuration statistics *below*. The asterisk by the *AeAlbo* AalbF2 assembly indicates that the early stage assembly annotation has a redundant list of gene models.

Because many viruses replicate their RNA genomes via a double-stranded RNA (dsRNA) intermediate, the conserved RNA interference (RNAi) pathway provides antiviral activity through Dicer and Argonaute enzymes converting viral dsRNA into siRNAs for repressing viruses (Samuel et al. 2018; Guo et al. 2019). Recently, the piRNA pathway was also implicated in assisting the siRNA pathway with antiviral response in the culicine mosquitoes and cell culture lines (Goic and Saleh 2012; Olson and Blair 2015; Halbach et al. 2017; Lambrechts and Saleh 2019).

A key knowledge gap is the degree to which viral siRNAs and piRNAs make up or affect mosquito small RNA transcriptomes. Previous mosquito studies have mainly focused on either virus derived small RNAs (Myles et al. 2008, 2009; Sánchez-Vargas et al. 2009; Brackney et al. 2010; Scott et al. 2010; Hess et al. 2011; Morazzani et al. 2012; Saldaña et al. 2017; Varjak et al. 2017a,b; Rückert et al. 2019) or conducted genomic analyses on earlier incomplete assemblies and preliminary annotations of individual mosquito species (Akbari et al. 2013; Whitfield et al. 2017; Tassetto et al. 2019). In this study, we generated more than 50 new small RNA libraries from cell cultures, male and female gonads, and respective carcasses from four medically important mosquito species (*AnGam*, *CuQuin*, *AeAeg*, *AeAlbo*) to add to the trove of publicly available small RNA libraries. We then implemented our small RNA analysis pipeline to enable cross-species comparisons. Our analysis provides the first comprehensive view of small RNA transcriptomes across mosquito phylogeny, reveals novel evolutionary and host dynamics in viral and somatic piRNA production, and uncovers notable periodicity in phased piRNA biogenesis patterns within culicine mosquitoes.

## Results

### Framework for integrated small RNA analysis across four mosquito species

We previously built functional annotation pipelines for small RNA libraries generated from the gonads of Drosophilids, mammals, and other vertebrates (Chirn et al. 2015). To extend this pipeline to compare small RNAs across mosquito genomes (Fig. 1B), we added a curated list of arboviruses. We queried NCBI GenBank for mosquito arboviruses and viral gene names (Nanfack Minkeu and Vernick 2018; Zakrzewski et al. 2018) and the Virus Pathogen Resource (VIPR) (Pickett et al. 2012) to make a list of 225 mosquito arboviruses in May 2019 that exceeds the 107 Drosophilid viruses listed in Palmer et al. (2018). We manually inspected entries to reduce redundancy among similar entries that are just slight sequence variants of a single virus class.

Our study took advantage of new genome assemblies of various culicine mosquito species and additional genome annotation resources from the legacy VectorBase database (Holt et al. 2002; Nene et al. 2007; Arensburger et al. 2010; Bartholomay et al. 2010; Giraldo-Calderón et al. 2015). *AeAeg* and *AeAlbo* genome assemblies were enhanced with Hi-C information and longer reads sequencing to connect scaffolds into chromosomal assembles (Dudchenko et al. 2017; Matthews et al. 2018; Palatini et al. 2020). From these assemblies, the transposon consensus sequences list were processed to reduce redundancy (Supplemental Fig. S1**;** Supplemental Materials). Last, we curated viruses and transposon consensus lists (Supplemental Files 1–7) and the compendium of outputs in a publicly accessible database resource of mosquito small RNA genomics (MSRG; https://laulab.bu.edu/msrg/).

MSRG outputs are organized by the four individual species, with species-specific results described in the Supplemental Text and in Supplemental Figure S2 (*AnGam*), Supplemental Figure S3 (*CuQuin*), Supplemental Figure S4 (*AeAeg*), and Supplemental Figure S5 (*AeAlbo*). These full galleries show complete species-focused analyses of endogenous and arboviral small RNA functional classes and features. The standard culture conditions for the mosquito cells profiled in this study are described in Supplemental Table S1, whereas the sequencing statistics of the libraries analyzed per species as well as the curated lists of genic and intergenic piRNA-containing loci are in Supplemental Table S2 (*AnGam* Metatable), Supplemental Table S3 (*DMel* Metatable), Supplemental Table S4 (*CuQuin* Metatable), Supplemental Table S5 (*AeAeg* Metatable), and Supplemental Table S6 (*AeAlbo* Metatable). These outputs enabled comparison between samples and species libraries to derive insights into virus- and transposon-targeting features by the mosquito small RNA transcriptomes.

### Multiple common arboviruses persistently infect and generate small RNAs in mosquito cell cultures

**Figure 2.**
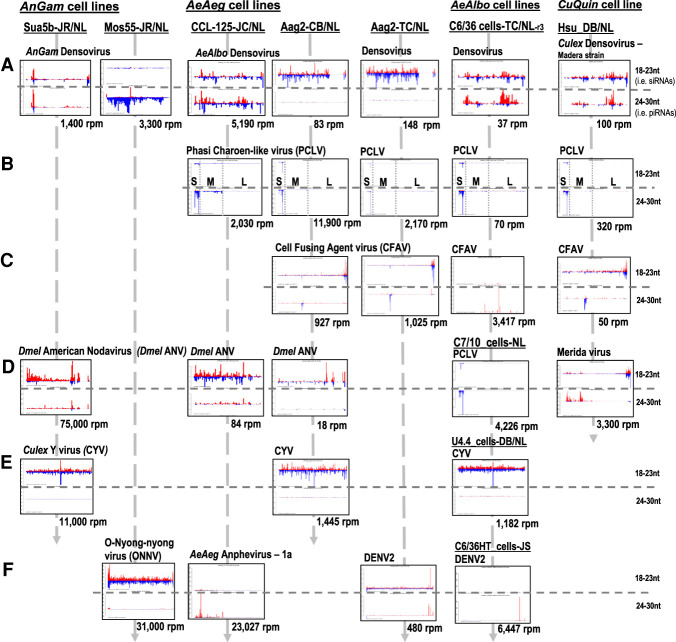
Multiple arboviruses persistently infect mosquito cell cultures and generate arboviral small RNAs. Profiles of viral small RNAs in cell culture lines from *AnGam*, *CuQuin*, *AeAeg* and *AeAlbo.* Reads per million (rpm) numbers are totals of the siRNA-length and piRNA-length small RNAs that come from the plus strand in red and minus strand in blue. The *x*-axis gives the coordinates of the virus sequence, and the *y*-axis is the autoscaled read frequency. The total small RNA normalized counts are *below* each plot. The suffix to sample names is the initials of the laboratory investigator where the sample was originally obtained: (JR/NL) Jason Rasgon to Nelson Lau; (DB) Doug Brackney; (JC) John Connor; (CB) Carol Blair; (TC) Tonya Colpitts; (JS) Juan Salas-Benito. The S, M, and L segments of the Phasi Charoen-Like virus (PCLV) are marked on these coverage plots. (*A*) Various species densoviruses. (*B*) Phasi Charoen-like virus. (*C*) Cell fusing agent virus. (*D*) *Drosophila* American nodavirus and two other cells with PCLV and Merida virus. (*E*) *Culex* Y virus. (*F*) Alphaviruses and flaviviruses.

Because many mosquito cell cultures were generated decades ago (Supplemental Table S1), we expected they would carry viral small RNAs from persistent arbovirus infections (Fig. 2). However, specific arboviruses could also infect across multiple Dipteran species. For example, consistent with earlier reports (Chandler et al. 2014; Zhang et al. 2016; Maringer et al. 2017; Di Giallonardo et al. 2018; Weger-Lucarelli et al. 2018), there was broad distribution of Phasi Charoen-like virus (PCLV) and Cell Fusing Agent virus (CFAV) viral piRNAs among different species of culicine mosquito cell lines (Fig. 2B,C). We also detected viral small RNAs in the *AnGam* Sua5b-JR line and the *AeAeg* CCL-125-JC and Aag2-CB lines from the *Drosophila* American nodavirus (*Dme*l ANV; related to Flock House virus or FHV) (Fig. 2D) that persistently infects *Drosophila* Schneider 2 (S2) line and OSS cells (Aliyari et al. 2008; Flynt et al. 2009; Wu et al. 2010; Han et al. 2011). In addition, abundant viral siRNAs from *Culex* Y virus (CYV) were in *AnGam*, *AeAeg*, and *AeAlbo* cell lines (Fig. 2E). These data support the broadness of these arbovirus tropisms spanning these Dipteran species.

The *AeAeg* densovirus is a small single-stranded virus previously developed for gene transduction of mosquitoes and mosquito cell cultures (Afanasiev et al. 1994, 1999). Our analyses revealed densoviral siRNAs and piRNAs across many cell lines except for the *AeAlbo* C7/10 and U4.4 cells (Fig. 2A). We detected abundant antisense densoviral piRNAs in the *AnGam* Mos55-JR line (-JR from the Rasgon laboratory) versus no densoviral small RNAs in the Mos55-TC line (-TC from the Colpitts laboratory), yet both displayed a persistent infection of densovirus (Supplemental Fig. S6A), suggesting that densovirus genome integration enables Mos55-JR to generate the densoviral piRNAs. Persistent densovirus infections in C6/36 cells had been proposed to enable stable coinfections with DENV2 (Burivong et al. 2004; Kanthong et al. 2008), suggesting a selective advantage for cells to harbor densovirus.

Recently, persistent infections of mosquito cell cultures by pathogenic arboviruses like flaviviruses and alphaviruses have been reexamined (Avila-Bonilla et al. 2017; Fredericks et al. 2019; Koh et al. 2019; Reyes-Ruiz et al. 2019). Among our mosquito cell cultures, we also discovered persistent viral infections reflected by abundant viral siRNAs against ONNV in the Mos55-JR line, and DENV2 siRNAs and piRNAs in the Aag2-TC line (Fig. 2F). Perhaps similarly to how *Dmel* ANV may have passed between *Drosophila* cells to mosquito cells, these infections were most likely inadvertent. Last, abundant viral piRNAs from *AeAeg* Anphevirus-1a were detected in the CCL-125-JC line but not in our Aag2 cells, which are reported to also be persistently infected (Di Giallonardo et al. 2018; Parry and Asgari 2018), reflecting the similar dichotomy of persistent densovirus in both Mos55 cell strains but densoviral piRNAs only expressed in one of the strains.

### Higher levels of somatic piRNAs in mosquitoes with persistent arboviral small RNAs

**Figure 3.**
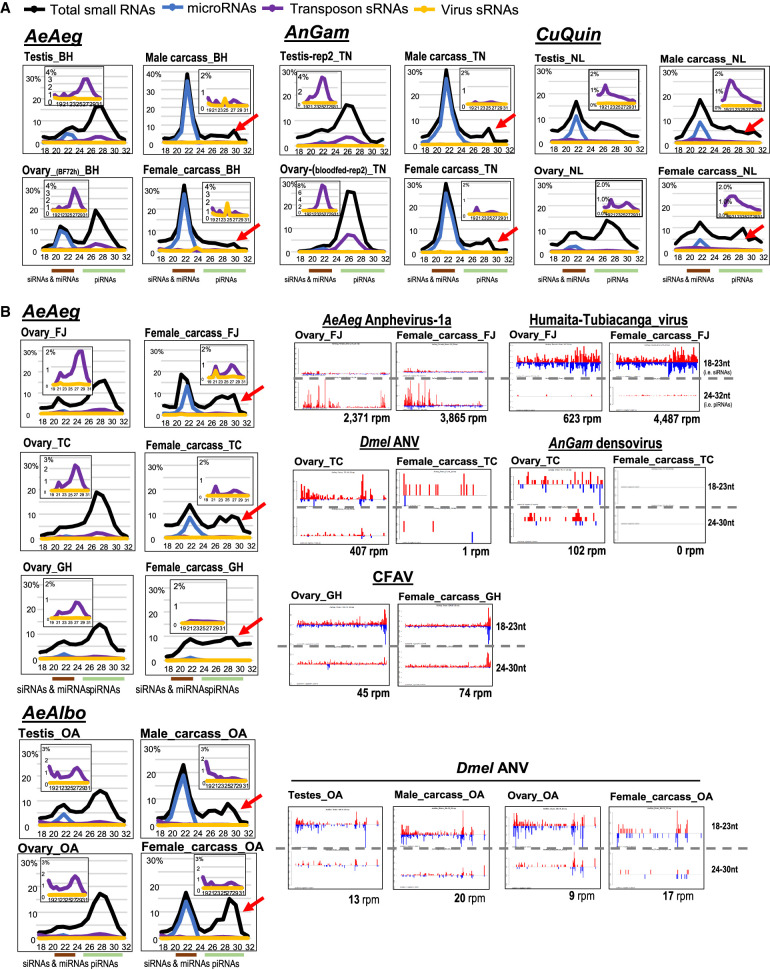
Variation in proportion of somatic piRNAs in mosquito strains correlates with persistent arboviral small RNAs. (*A*) Small RNA size distributions from mosquito samples in which the somatic piRNA levels are much lower in comparison to the gonads, and these samples lack other arbovirus small RNAs. Colored lines at the *bottom* mark the siRNAs and miRNAs ranging between 19 and 23 nt, whereas piRNAs are between 24 and 30 nt. The *inset* charts magnify the distribution of transposon and virus sRNAs under a different *y*-axis range, and the red arrow points to low levels of somatic piRNAs. (*B*) Additional small RNA size distributions (*left*) of mosquito samples with high levels of somatic piRNAs along with the detection of other persistent arbovirus small RNAs in the pattern plots (*right*). The *x*-axis gives the coordinates of the virus sequence; the *y*-axis is the autoscaled read frequency. The total small RNA normalized counts are *below* each plot.

Animal piRNAs mainly silence transposons in gonads to ensure fertility, with less evidence for somatic functions in mammals where somatic piRNAs are lowly expressed. However, mosquitoes are like most other insects expressing significant somatic piRNAs, and only *Drosophila* is the outlier for low levels of somatic piRNAs (Lewis et al. 2018; Genzor et al. 2019). In spite of this, some mosquito carcasses had subdued amounts of somatic piRNAs (*AeAeg*, female and male carcasses from BH; *AnGam*, male and female carcasses from TN; and *CuQuin*, male and female carcasses from NL) (Fig. 3A). This contrasted other mosquito carcasses containing abundant somatic piRNAs (*AeAeg*, female carcasses from FJ, TC, and GH; and *AeAlbo*, male and female carcasses from OA) (Fig. 3B).

What could explain this variation of somatic piRNA levels among different isolates of the same species of *AeAeg*? We ruled out unintended detection bias like residual gonads contaminating the carcass, because there were no contaminating germline transcripts like *vasa*. We then hypothesized that three *AeAeg* isolates with abundant somatic piRNAs may be a result of persistent arbovirus infection as reflected by viral small RNAs. This hypothesis was supported by the absence of viral small RNAs in the *CuQuin* samples we analyzed, the *AeAeg* isolate from the Hay laboratory (Akbari et al. 2013) and the *AnGam* isolate from the Nolan laboratory (this study; Castellano et al. 2015).

Indeed, our analysis showed that *AeAeg* isolates with abundant somatic piRNAs also carried persistent arbovirus infections reflected by viral small RNAs (Fig. 3B). The FJ *AeAeg* strain from Miami, FL (Lewis et al. 2018) expressed *AeAeg* Anphevirus strain-1a piRNAs and viral siRNAs from the Humaita-Tubiacanga virus (HTV), similar to HTV siRNAs detected in *AeAeg* strains from Rio de Janeiro, Brazil (Aguiar et al. 2015). In the *AeAeg* TC isolate of the ROCK strain, we detected *Dmel* ANV siRNAs and densovirus small RNAs. Last, the GH *AeAeg* strain from Galveston, TX, harbored persistent CFAV (Kim et al. 2009) and both CFAV siRNAs and piRNAs in the ovary and carcass (Fig. 3B).

Somatic piRNA levels were also high in the OA *AeAlbo* strain from Los Angeles, CA (Gamez et al. 2020), which correlated with persistent *Dmel* ANV (Fig. 3B). Other reports have described *AeAlbo* viral small RNAs from densovirus (Morazzani et al. 2012) and ONNV (Wang et al. 2018), which are circulating in wild mosquito populations. We speculate the *Drosophila* laboratory stocks, a reservoir for nodaviruses (Goic et al. 2013; Kandul et al. 2019) could explain these Drosophilid arboviruses persisting in *AeAlbo* strains (Supplemental Fig. S5E).

### Potential cross talk between flavivirus infection and endogenous small RNA levels

Despite wide competency of *AeAeg* cells and mosquitoes to support arbovirus replication, viral piRNAs are a minor fraction of total small RNAs, even with ectopic infections of CHIKV, DENV, or ZIKV (<6%) (Fig. 3A,B; Supplemental Fig. S4A,B). *AeAeg* mosquitoes and cell cultures appear unaffected by arbovirus infection presumably because antiviral RNAi pathways are generating viral siRNAs and piRNAs (Aliyari and Ding 2009; Karlikow et al. 2014; Blair and Olson 2015; Samuel et al. 2018). However, new infections from pathogenic viruses can be affected by persistent infections of other arboviruses, perhaps through small RNA cross talk (Burivong et al. 2004; Kanthong et al. 2008, 2010; Myles et al. 2008; Goic and Saleh 2012; Parry and Asgari 2018; Reyes-Ruiz et al. 2019).

**Figure 4.**
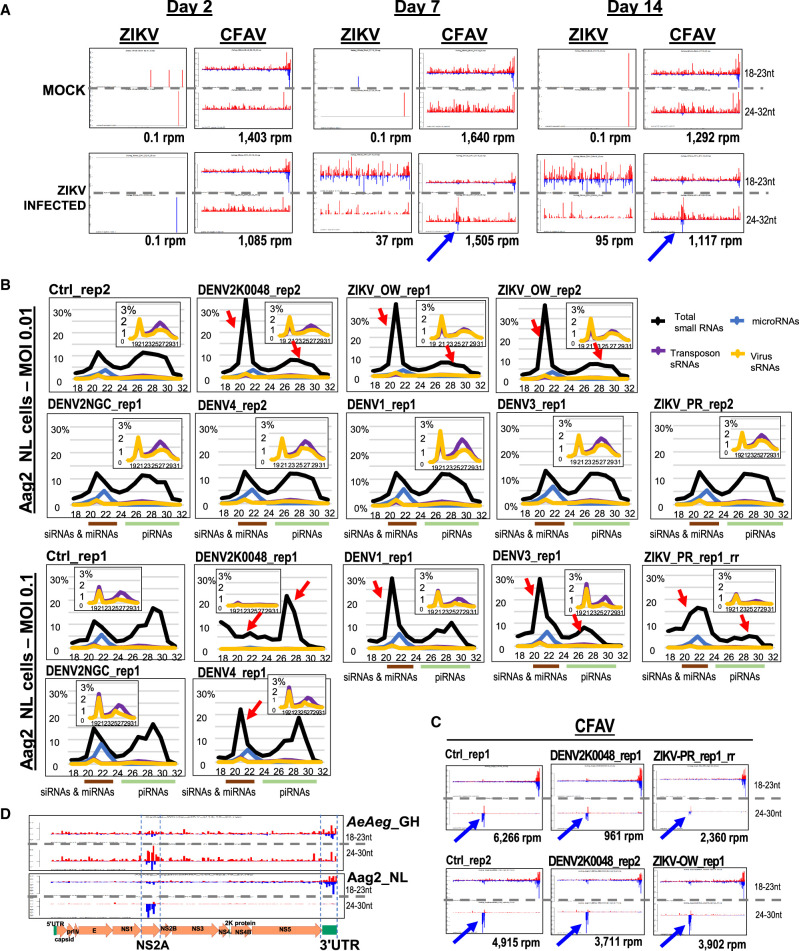
Small RNA cross talk in *Aedes aegypti* (*AeAeg*) during flavivirus infections. (*A*) Reanalysis of ZIKV and CFAV small RNAs from *AeAeg* females as sequenced from Saldaña et al. (2017). The blue arrow notes emergence of new piRNAs from CFAV after active replication of ZIKV small RNAs. The *x*-axis gives the coordinates of the virus sequence; the *y*-axis is the autoscaled read frequency. The total small RNA normalized counts are *below* each plot. (*B*) Small RNA length distributions as a proportion of the small RNA library. The *inset* graph zooms in on the modest proportions of viral and transposons small RNAs. Red arrows point to the significant change from the normal proportion of small RNAs in control cells. (*C*) Counts and small RNA profiles from CFAV in control and infected Aag2-NL cells. Blue arrows point to preexisting group of negative strand piRNAs potentially because of multiple preexisting viruses replicating and generating small RNAs in Aag2-NL cells. (*D*) The regions generating notable piRNAs and siRNAs from CFAV in mosquitoes and Aag2 cells are the NS2A gene and 3′ UTR.

To see if flavivirus infection affected endogenous small RNA levels in mosquitoes and cell cultures, we reanalyzed small RNAs from female *AeAeg* mosquitoes fed blood that lacked or contained ZIKV. We reconfirmed that both ZIKV siRNAs and piRNAs were only detectable 7- and 14-d post-infection (Saldaña et al. 2017). Whereas bulk overall small RNAs were the same whether the mosquitoes harbored ZIKV or not (Supplemental Fig. S7A), our analysis revealed new piRNAs from a specific region of CFAV only stimulated after ZIKV replication (Fig. 4A, blue arrows). This region did not have specific homology with ZIKV piRNAs but generated both plus and minus-strand piRNAs indicative of the “ping-pong” mode of piRNA interactions. Despite clear signals of ZIKV and CFAV small RNAs, these viral small RNAs were only a tiny fraction of the total small RNA samples in these libraries (Supplemental Fig. S7A).

Next, we tested if flavivirus infections of Aag2 cells with DENV and ZIKV might also affect CFAV small RNA patterns. Therefore, we performed DENV and ZIKV infections at two different multiplicities of infection (MOI, 0.1 and 0.01) of Aag2 cells, including two strains of the DENV2 serotype (NGC-a high passage and K0048-low passage) (Troupin et al. 2016); as well as the Old World (OW) and Puerto Rico (PR) isolates of ZIKV (Fig. 4B; Araujo et al. 2020). Because the Aag2 cells were incubated for 7 d post-inoculation, the higher MOI = 0.1 left fewer cells and viral RNAs remaining compared to the lower MOI = 0.01 (Supplemental Fig. S7B).

Flavivirus small RNAs correlated with viral genomic RNA levels measured by qRT-PCR, but there was variability in the proportions of flavivirus siRNAs and piRNAs (Supplemental Fig. S7C). The unusual patterns of abundant singular DENV piRNAs from the plus strand that we observed was consistent with other studies (Scott et al. 2010; Hess et al. 2011; Miesen et al. 2016). Whereas DENV and ZIKV siRNAs were generated from both plus and minus strands indicative of a dsRNA precursor, the viral piRNAs were biased from the plus strand and predominantly arose from a few very abundant reads (Supplemental Fig. S7D, bottom plots), recapitulating the same confounding patterns observed by others (Goic et al. 2016; Miesen et al. 2016; Whitfield et al. 2017; Merkling et al. 2020). This pattern of viral piRNA accumulation defies the generalized biogenesis patterns of phased piRNAs (Han et al. 2015; Mohn et al. 2015; Pandey et al. 2017; Gainetdinov et al. 2018; Izumi et al. 2020).

Although both batches of Mock Control Aag2 cells had expected bimodal distributions of 18–23 nt siRNAs and miRNAs versus 24–32 nt piRNAs, we observed instances in which these distributions were greatly affected by viral infection. In both replicates, DENV2K0048 distorted these two distributions, in one case greatly enhancing endogenous siRNAs while depressing piRNAs, and in another case a vice versa response (Fig. 4B, red arrows). Also, in both replicates, the ZIKV_OW infections enhanced endogenous siRNAs while depressing piRNAs, but this was vice versa in one ZIKV_PR infection. Although DENV2NGC, the high passage strain, repeatedly lacked impact on small RNA populations, there was marked variability in one of the experiments but not in the other for when DENV1, DENV3, and DENV4 infections greatly affected the bimodal distribution of piRNAs versus siRNAs and miRNAs.

Future studies will dissect this variability in the small RNA populations of Aag2 cells during arbovirus infection. However, two batches of Mock Control Aag2 cells already displayed enhanced minus-strand piRNAs similar to the region of CFAV piRNAs amplified in the ZIKV-infected mosquitoes (Fig. 4C). Because Aag2 cells are already persistently infected by multiple arboviruses, the DENV and ZIKV infections did not affect these CFAV piRNAs corresponding to the NS2A gene (Fig. 4D). What specifies the NS2A gene as a piRNA precursor and CFAV 3′ UTR as a stronger initiator of siRNA biogenesis remains unclear (Fig. 4A), although other flavivirus 3′ UTRs have been described to have an antiviral role (Moon et al. 2015).

### Repetitive element targeting by endogenous piRNAs

**Figure 5.**
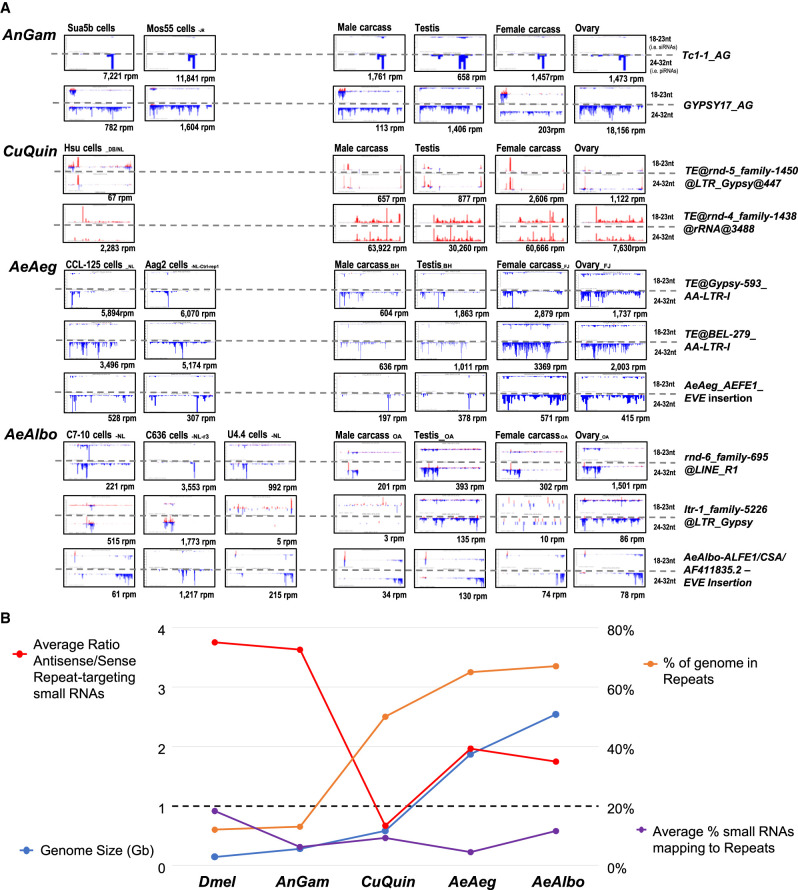
Transposons and repeats are targeted by common small RNAs in mosquito cells and tissues. (*A*) Profiles of the transposons and repeats with the most abundant small RNAs both in cell cultures and mosquito tissues. Positive strand reads are in red; minus-strand reads are in blue. The *x*-axis gives the coordinates of the transposon and repeats sequence; the *y*-axis is the autoscaled read frequency. The total small RNA normalized counts are *below* each plot. (*B*) Comparisons of Dipteran genome sizes, fraction of the genome as repeats, average percentage of the small RNAs targeting mosquito transposons and repeats, and the average ratios of the repeat-targeting small RNAs being antisense or the same sense as the repeats.

Mosquito genomic insertions called Endogenous Viral Elements (EVEs) were proposed to have an antiviral role by generating endogenous piRNAs complementary to flavivirus sequences (Katzourakis and Gifford 2010; Lequime and Lambrechts 2017; Suzuki et al. 2017; Whitfield et al. 2017; Houé et al. 2019; Tassetto et al. 2019; Blair et al. 2020). The most active EVE in our data set, the AEFE1/AY347953 EVE has homology with the NS5 gene of flaviviruses like Kamiti River virus and CFAV (Crochu et al. 2004) and predominantly generated piRNAs with fewer siRNAs in the gonads, soma, and cell lines (Fig. 5A). In contrast, antisense piRNAs to PCLV, largely from the S-fragment of the PCLV genome (Fig. 2B), suggests this is also an EVE signature (Whitfield et al. 2017; Tassetto et al. 2019). *AeAeg* mosquitoes and cell cultures produced significant CFAV small RNAs from the CFAV-like EVE, which should theoretically target CFAV (Figs. 2C, 3B; Suzuki et al. 2017; Whitfield et al. 2017), yet there is persisting replication of CFAV RNAs in the GH *AeAeg* isolate (Fig. 4A). We were unable to cross-reference other analyses of *AeAeg* EVEs (Whitfield et al. 2017; Tassetto et al. 2019) because this was performed on an incomplete genome assembly from their isolate of the Aag2 cell line. In summary, EVEs may be contemporary versions of the more ancient LTR-containing transposons that are templates for abundantly generating small RNAs.

Among the other most prominent mosquito transposons to generate piRNAs in both cell cultures and animals were LTR-containing transposons, along with notable LINE-like retrotransposons and the Tc1 DNA-type transposon in *AeAlbo* and *AnGam*, respectively (Fig. 5A). There were also cell line–specific and soma-versus-germline differences in small RNA targeting of transposons, with the greatest number of transposons with small RNA targeting evident in the germline tissues (clustering heatmaps and coverage plots in Supplemental Figs. S2E,F, S3E,F, S4F,H, S5E,I).

Piwi proteins require antisense piRNAs to target transposon sense transcripts (Post et al. 2014; Batki et al. 2019), so we expected *Drosophila* small RNAs to have a biased ratio of ∼3.8:1, antisense:sense mapping to transposons (Fig. 5B). Although *AnGam* had a lower fraction of small RNAs mapping to transposons than *Drosophila* (∼6% vs. ∼18%), the culicine mosquitoes had the lowest proportion of small RNAs mapping antisense to transposons. In fact, *CuQuin* small RNAs were slightly biased for sense mapping reads to repeats such as the top examples of an LTR-Gypsy transposon and rDNA repeats small RNAs (Fig. 5A,B). Although we cannot explain this *CuQuin* discrepancy, other differences in our transposon piRNA quantitation, such as *AeAlbo* piRNAs measured in Liu et al. (2016), can be attributed to using the newer *AeAlbo* assembly (Palatini et al. 2020) and reducing the redundancy in repeat lists (Supplemental Fig. S1).

**Figure 6.**
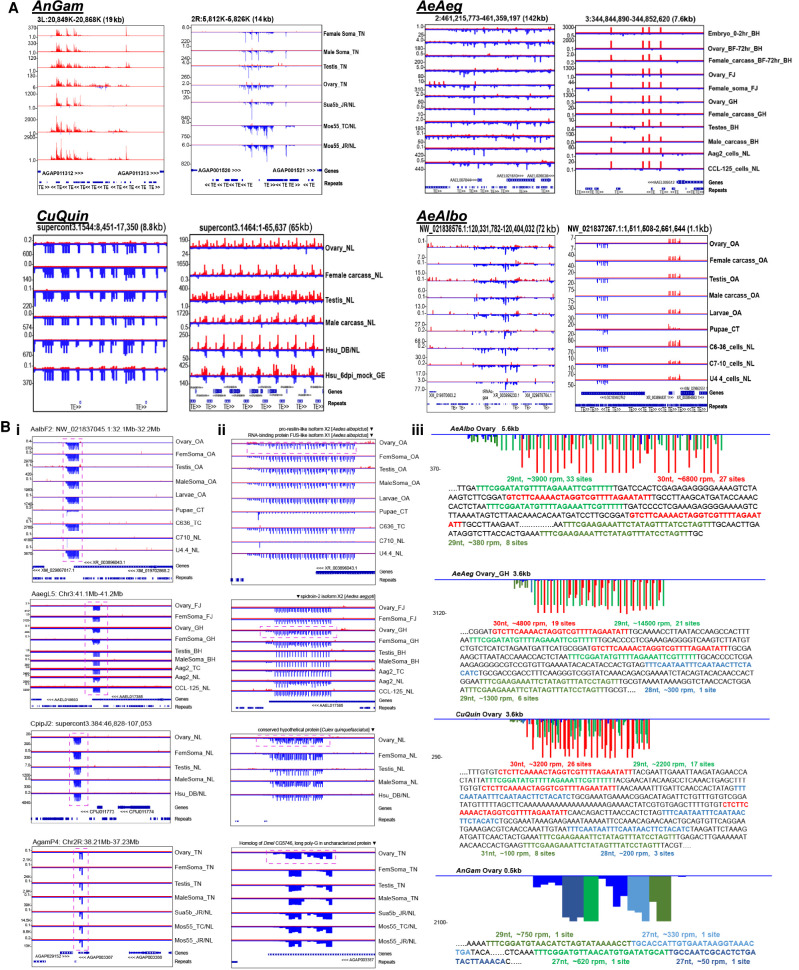
Prominent mosquito piRNA cluster loci. (*A*) Genome browser snapshots of notably large piRNA Cluster Loci (piRCL) in mosquitoes. Genes and repeats (TEs) tracks are at the *bottom* of each snapshot. (*B*) A dynamically evolving Mosquito-Conserved piRNA Cluster locus (MCpiRCL) expressed throughout gonads, soma, and cell cultures. (*i*) Zoomed out genome browser snapshots at the kilobase level of the MCpiRCL. (*ii*) Zoomed in view of the MCpiRCL from the dashed box in (*i*). The descriptions of the nearest transcript are listed at the *top* of the browser window. (*iii*) Microscopic view of the MCpiRCL from the dashed box in (*ii*). The peaks are color-coded according to the specific reads as DNA in the sequence *below* each diagram, derived from the region highlighted by the dashed box *above* the sequence. Reads per million (rpm) and how many occurrences of the read in the satellite tandem repeats within this MCpiRCL.

For *Drosophila* to generate piRNAs antisense to transposons, the transposon sequences in major piRNA cluster loci (piRCL) are oriented antisense to the single plus strand precursor transcript like in the *flamenco* locus (Li et al. 2009; Malone et al. 2009). Although *flamenco* homologs are only conserved in the closest relatives of *D. melanogaster* (Chirn et al. 2015), *flamenco* is notable for its high unistrand expression of piRNAs in the somatic compartment of *Drosophila* follicle cells and dense insertions of transposons and repeats. Only a few instances of the largest piRCLs in mosquitoes display similar features of unistrand piRNA expression both in the germline and soma proper (Fig. 6A; Supplemental Figs. S2–S5; Supplemental Tables S2–S6). However, in contrast to *Drosophila flamenco*, the transposon density in these “*flamenco*-like” mosquito piRCLs appears lower and with fewer piRNAs directly overlapping transposon sequences (Fig. 6A). One of our determinations was also confirmed by the Marques laboratory annotation of a “*flamenco*-like” cluster in *AeAeg* (Aguiar et al. 2020), and through genome synteny, we found a homologous piRCL in *AeAlbo*, but it is half the size of its counterpart in *AeAeg* (∼72 kb vs. ∼142 kb) (Fig. 6A). These observations underlie the dynamic evolution of these piRCLs among mosquitoes.

### A major genic piRCL is dynamically evolving yet syntenically conserved through mosquito phylogeny

To define other genic and intergenic piRCLs in mosquitoes (Supplemental Tables S2, S4–S6), we combined automated genome scanning with manual curation. The six top major *AeAlbo* piRCLs exist on three superscaffolds, with mostly single-stranded biases in the small RNA expression patterns (Supplemental Fig. S5J,K). Two of these *AeAlbo* genic piRCLs displayed patterns of satellite DNA repeats (Supplemental Fig. S5J, rightmost windows), which we also observed in other *CuQuin* and *AeAeg* piRCLs with satellite DNA repeats generating very abundant amounts of piRNAs (Supplemental Figs. S3H, S4J) but no such satellite DNA repeats in *AnGam*. In addition, the lack of synteny around these piRCLs made it challenging to compare these particular piRCL across the mosquito species.

However, one *AeAlbo* piRCL with satellite DNA repeats enabled comparative genomics because it was linked to protein-coding genes (Fig. 6Bii). Expressed very highly in *AeAlbo* gonads, somatic tissues, and cell cultures, this genic piRCL generates on average more than 10,000 reads per million (rpm) from mainly two major piRNAs which have 33 and 27 alternating repeats spread out in a ∼5.6 kb region (Fig. 6Biii). The *AeAeg* orthologous gene also contained a genic piRCL with satellite DNA repeats and identical piRNA sequences, but a different arrangement of 21 and 19 alternating repeats (Fig. 6Biii, second row).

The orthologous *CuQuin* genic piRCL also displayed satellite DNA repeats with two alternating piRNA sequences from 17 and 26 repeats abundantly expressed in gonads, somatic tissues, and the Hsu cell line (Fig. 6Bii, third row). One satellite piRNA's primary sequence, UUUCGGAUAUGUUUUAGAAAUUCGUUUUU, is perfectly conserved across mosquito evolution (Fig. 1A), but its repeat number has evolved from 17 sites in *Culex* to 21 and 33 sites in *Aedes* species. Notably, the other *Culex* satellite piRNA sequence differs from the *Aedes* sequence only by the first nucleotide of 5′-C in *Culex* and 5′-G in *Aedes* in each of 26 repeats in *CuQuin* versus the 19 and 27 sites in *AeAeg* and *AeAlbo*, respectively (Fig. 6Biii). The most parsimonious explanation for this type of sequence evolution is a base change first in the early divergence of their ancestors and then parallel evolutionary expansion of the mutated piRNA sequence to form these satellite DNA repeats.

In accordance with the long divergence between culicine and anopheline mosquitoes, *AnGam* appears to lack piRCLs containing satellite DNA repeats, however the orthologous genic piRCL extends to the *AnGam* gene *AGAP003387* (Fig. 6B, fourth row). In contrast to the culicine genic piRCL, this *AnGam* piRCL is very compact at ∼500 bp long within the 3′ UTR of *AGAP003387* with no tandem repeats but has four main piRNAs comprising >1500 rpm. Two of these *AnGam* piRNAs were perfectly conserved at the primary sequence level as one of the culicine satellite DNA piRNAs (Fig. 6Biii), and this *AnGam* piRCL was also abundantly expressed in *AnGam* gonads and cell cultures. The gene *AGAP003387* only has homologs within other mosquitoes, whereas a neighboring gene *AGAP003388* is homologous to the *Dmel* gene *CG5746* that does generate some 3′ UTR piRNAs (Chirn et al. 2015). Therefore, we have named this a Mosquito-Conserved piRNA Cluster Locus (MCpiRCL).

The *AnGam* piRCL may represent the ancestral mosquito locus more than about 200 MYA that began as genic piRCL region already primed to express important piRNAs. As the culicine branch expanded their genomes with transposon repeats, the MCpiRCL also gained satellite DNA repeat perhaps to amplify piRNA expression. This satellite DNA piRCL was also discovered in *AeAeg* by (Halbach et al. 2020), and was proposed to cause maternally deposited transcripts to turn over during embryogenesis, similar to the vertebrate tandem repeat cluster of miRNAs miR-430 and miR-427 (Giraldez et al. 2006; Lund et al. 2009). However, whereas miR-430 and miR-427 expression is restricted to the embryo, the MCpiRCL in all four of these mosquitoes is expressed throughout the gonads, somatic tissues, and cell culture lines (Fig 6Biii), suggesting the targeting capacity of these piRNAs may be broader than maternally deposited transcripts. We predicted many hundreds of transcripts and highlight the top two mRNA, transposon, and virus targets in Supplemental Figure S8. Although the incomplete draft CpipJ2 genome assembly and annotation (Arensburger et al. 2010) may be limiting the number of predicted *CuQuin* targets, there is an expanded repertoire of potential gene and transposon targets for the *AeAeg* and *AeAlbo* piRNAs from this MCpiRCL.

### Culicine mosquitoes show periodicity in the patterns of piRNA biogenesis

Only culicine mosquitoes contained piRCL with satellite DNA repeats (Fig. 6B; Supplemental Figs. S3H, S4J, S5J), and these single abundant piRNAs were biased on one strand and spaced out from each other by a >29 nt gap. This piRCL configuration challenges the prototypical phasing pattern of primary piRNA biogenesis first described in *Dmel* (Han et al. 2015; Mohn et al. 2015; Pandey et al. 2017; Gainetdinov et al. 2018; Izumi et al. 2020). Indeed, a previous study applying piRNA phasing algorithms across piRNA data sets from a phylogenetic spectrum of hydra to insects to mammals showed that *AeAeg* piRNAs stood out with the most periodic of 5′ to 5′ piRNA distance peaks (Gainetdinov et al. 2018).

**Figure 7.**
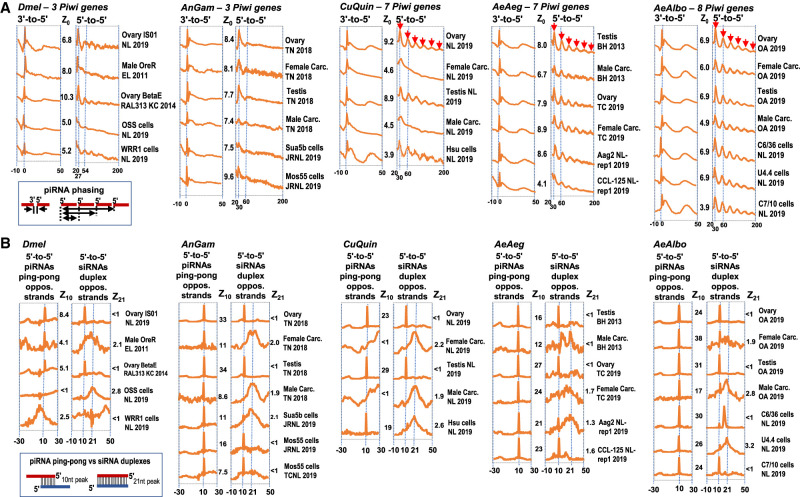
Mosquitoes with expanded Piwi pathway gene numbers display periodic piRNA biogenesis phasing patterns. (*A*) Autocorrelation analysis of the 3′-to-5′ and 5′-to-5′ piRNA phasing patterns from various small RNA libraries in the MSRG. Red arrows mark the periodicity of the 5′-to-5′ phasing in samples from independent laboratories and support a biological process rather than a technical feature in the detection of this periodic pattern. (*B*) Autocorrelation analysis of the piRNA ping-pong and overlapping siRNA patterns from various small RNA libraries in the MSRG, with Z_10_ and Z_21_ scores >1.0 as denoting a significant ping-pong piRNA or fully duplexed siRNA signature, respectively. The full gallery of additional pattern diagrams is in Supplemental Figure S10. The *x*-axis gives the base coordinates from the autocorrelation analysis, whereas the *y*-axis shows arbitrary units that vary for each individual library.

We applied the same algorithm of a LOWESS nonparametric regression and autocorrelation smoothing (Gainetdinov et al. 2018) to a wide number of *Dmel*, *AnGam*, *CuQuin*, *AeAeg*, and *AeAlbo* libraries. We confirmed the strong conservation throughout Dipterans of the one piRNA phasing mechanism that juxtaposes the 3′ terminus of the upstream piRNA to the 5′ start of the downstream piRNA (Fig. 7A; Supplemental Fig. S9). There was also a very periodic 5′-to-5′ phasing pattern for the *CuQuin*, *AeAeg*, and *AeAlbo* samples, both in mosquito tissues and cell cultures (Fig. 7A). However, this periodic pattern was dampened in *AnGam* and *Dmel*, with perhaps only *Dmel* ovarian small RNAs subjected to beta-elimination showing the enhanced periodic signal (Song et al. 2014).

We speculate the expansion of Piwi pathway genes in culicine mosquitoes (Lewis et al. 2016) may promote periodicity in piRNA phasing biogenesis patterns while also enabling the innovation of satellite DNA repeats in piRCL. To reexamine the evolutionary relationships of Dipteran Piwi pathway genes, we took *Dmel* Piwi pathway genes and conducted BLASTP and manual curation between NCBI GenBank and VectorBase to better define the mosquito homologs (Supplemental Table S7). Ten core Piwi pathway genes in *Dmel* had single orthologs in *AnGam* that were then expanded into multiple homologs in culicine lineages (Supplemental Fig. S10A; Supplemental Table S8). *AeAlbo* stands out from *AeAeg* and *CuQuin* with the most expanded Piwi pathway gene families including two *Ago3* homologs and three homologs of *valois* and *vreteno* (Supplemental Fig. S10A). Another 15 Piwi pathway genes from *Dmel* had single orthologs in mosquitoes (Supplemental Fig. S10B). Perhaps the expansion of *piwi*/*aub* homologs in culicine mosquitoes explains piRCL innovation such as *AeAeg* PIWI4 being required for the satellite repeat MCpiRCL (Halbach et al. 2020). Although seven *Dmel* genes in *Drosophila's* piRNA-mediated transcriptional silencing pathways (i.e., *panx*, *rhi*, *del*, and *cuff*) (Le Thomas et al. 2014; Mohn et al. 2014; Zhang et al. 2014) were completely absent in mosquito genomes, this may foretell potential mosquito-specific factors required for its unique repertoire of Piwi pathway genes.

Last, to examine whether more Piwi pathway genes in culicine mosquitoes might impact piRNA “ping-pong” biogenesis mechanisms, we adapted the autocorrelation algorithm to count the frequencies of 5′-to-5′ distances of piRNA reads mapping on the opposite strand, and then noted the Z_10_ scores > 2 as a signal that piRNA ping-pong signatures were significant (Fig. 7B). We also analyzed siRNA reads with this same algorithm but noting Z_21_ scores > 2 as a signal of siRNA duplexes processed by Dicer. The piRNA ping-pong signatures were strong in all mosquito cell culture lines and gonads, but the ping-pong signature present in the carcasses of *AnGam*, *AeAeg*, and *AeAlbo* were absent in *CuQuin* carcasses. In most of the mosquito carcasses and some of the cell lines, an siRNA duplex signature was evident. From these results, we interpret that piRNA ping-pong mechanisms and Dicer-generation of siRNA duplexes generally remain the same among these Dipterans.

## Discussion

Cell cultures are invaluable for genomic studies as shown by the important genomic, transcriptomic, and epigenetic data sets for model organism and human cell lines in the modENCODE and ENCODE Projects, respectively (Graveley et al. 2011; Kharchenko et al. 2011; Nègre et al. 2011; Djebali et al. 2012; The ENCODE Project Consortium 2012; Thurman et al. 2012). Mosquito cell cultures from various species (Fig. 1C) also facilitate virology studies, and our study can place cell lines in better context to the tissues of the animal. For example, our principal component analysis (PCA) plots (Supplemental Fig. S11) and hierarchical clustering of miRNA and transposon small RNA profiles show that cell cultures have distinct transcriptomes from gonads and somatic tissues (Supplemental Figs. S2D,E, S3D,E, S4E,J, S5G,H). However, the PCA plots also suggest that different laboratories’ isolates of *AnGam*, *CuQuin*, and *AeAeg* cell cultures showed a higher degree of clustering together than the cell lines from *AeAlbo*.

Mosquitoes have a major translational impact on human health, yet genomic characterizations of the culicine mosquitoes have lagged because their significantly larger genomes are inflated by repetitive elements. New genomic approaches such as high-throughput long-read and Hi-C sequencing may bridge scaffolding gaps to bring about major improvements in the *AeAeg* and *AeAlbo* genome assemblies (Dudchenko et al. 2017; Matthews et al. 2018; Palatini et al. 2020). However, functional annotations, such as improving gene models with better transcriptome data, are still needed for mosquito genomics advancement including this study, in which we opted to analyze the CpipJ2 assembly that had genes and repeats tables (Arensburger et al. 2010) but was still more fragmented than the newer CpipJ3 assembly which lacked annotation (Dudchenko et al. 2017). Our study also shows the need for better repetitive element annotations including refinement of transposons beyond the automated programs like RepeatModeler (Wheeler et al. 2013; Flynn et al. 2020), which generate comprehensive but redundant repeat lists. Notably, the majority of mosquito piRNAs across species do not appear to target transposons and may ultimately have a wide range of other targets yet to be determined.

As the diversity of *Dmel* cell culture lines has greatly expanded just in the last decade, only four *Dmel* lines are known to express piRNAs (fGS/OSS, OSS-OSCs-OSC-delta-MBT, WRR1 and Kc cells) (Lau et al. 2009; Saito et al. 2009; Fagegaltier et al. 2016; Sumiyoshi et al. 2016; Vrettos et al. 2017), whereas the vast majority of *Dmel* cell lines only express miRNAs and siRNAs (Wen et al. 2014). Such few piRNA-expressing *Dmel* cell lines may reflect the exceptional nature of *Dmel* to restrict Piwi pathway gene expression to the gonads, whereas most other insects robustly express piRNAs in the soma (Lewis et al. 2018). The smaller selection of mosquito cell lines (Supplemental Table S1) coupled with their long history would contribute to their gene expression profiles diverging greatly from mosquito tissues. Yet every mosquito cell line in this study expressed piRNAs, including our culture of C7/10 cells (Fig. 2) that may differ from a previous report of C7/10 cells that lacked piRNAs (Skalsky et al. 2010).

With this initial survey of cell cultures and wild-caught versus domesticated laboratory mosquitoes, our data suggest that somatic piRNAs and siRNAs may be an insect vector response to a persistent arbovirus infection. Our future effort is to profile more wild mosquito isolates as additions to the MSRG resource. In addition to mosquito field studies, the MSRG resource will enhance future virology and biochemistry of mosquito cell cultures. Last, the MSRG resource provides a reference list of curated mosquitoes genic and intergenic piRCLs (Supplemental Fig. S11C; Supplemental Tables S2, S4–S6) and reference lists of mosquito arboviruses and transposons with abundant small RNAs from both cell cultures and colonies, which will aid the direction of future functional genomics studies.

## Methods

### Mosquito strains, cell cultures, and virus infections

The *AnGam* isolate from Imperial College, UK, was kept in standard rearing conditions as in Castellano et al. (2015). The *AeAeg* isolates from Colpitts laboratory were maintained in the insectary of the National Emerging Infectious Disease Laboratory (NEIDL) as described in Araujo et al. (2020). The *AeAeg* isolate from the Hughes laboratory was maintained in the insectary at the University of Texas Medical Branch as described in Saldaña et al. (2017). The *AeAlbo* isolates from the Akbari laboratory were described in Gamez et al. (2020). The *CuQuin* isolates were purchased from Benzon Research.

All mosquito cell culture media are described in Supplemental Table S1, and all cultures were established in the Lau laboratory for months before cells were used for total RNA extraction and multiple live aliquots were cryopreserved. Cells were all kind gifts: Sua5b and Mos55 cells from the Rasgon laboratory; C6/36 and Mos55 cells from the Colpitts laboratory; Aag2 cells from the Blair laboratory and Colpitts laboratory; CCL-125 from the Connor laboratory; C7/10 cells from the Fallon laboratory; and U4.4 and Hsu cells from the Brackney laboratory. All cells were maintained in a humidified incubator at 28°C with 5% CO_2_ atmosphere. The DENV and ZIKV infections were performed on Aag2 cells that were ∼80% confluent in T25 flasks grown in Shield and Sang Media (Supplemental Table S1) using viral supernatants from previous C6/36 infections. The infections were conducted under two different multiplicities of infection (MOI = 0.1 and 0.01) in the BSL2+ facility in the NEIDL and were cultured for 7 d before cells were neutralized in the TRI-reagent for total RNA extraction. Viral infection status was confirmed by the qRT-PCR assay detailed in Araujo et al. (2020).

### Small RNA library preparation and deep sequencing

Most small RNA libraries were constructed from small RNAs size fractionated from Urea-Polyacrylamide Gel Electrophoresis as in Chirn et al. (2015), and only new *Dmel* libraries were subjected to a process Q-sepharose matrix enrichment of small RNAs (Srivastav et al. 2019). For size fractionation of small RNAs, 1–5 µg of total RNA from mosquito tissues and ∼10 µg of total RNA from cell lines was extracted with TRI-reagent. Size fractionation was performed on a urea-denaturing 15% polyacrylamide gel with TBE buffer and 18-nt and 32-nt fluorescent oligos were used as markers. Next, 18–32 nt sized RNA portion of gel was excised under UV and eluted in 500 µL 0.3M NaCl overnight with mild agitation at RT. Small RNA–containing eluate was saved and supplemented with two volumes of ethanol and 1 µL of 20 mg/mL glycogen for precipitation at −20°C overnight. Small RNAs were precipitated by centrifuging at 15,000 rpm for 20 min at 4°C. Small RNA–containing glycogen pellet was next washed with chilled 75% ethanol and eluted in 12 µL of freshly made 50% (w/v) PEG-8000 to enhance 3′ end ligation efficiency. Then, 6 µL of the small RNAs in PEG-8000 was used for library construction using NEBNext Small RNA Library Construction kit (E7330S) per the manufacturer's protocol.

All small RNA libraries were purified with the Monarch PCR & DNA Cleanup Kit (5 μg), quantified using Qubit 2.0, and analyzed on Agilent Bioanalyzer 2100 before sequencing on the BUSM Microarray and Sequencing Resource. For total RNA from *Drosophila* OSS and WRR1 cells and *AnGam* Sua5b and Mos55 cells, we subjected this to beta-elimination treatment as in Song et al. (2014).

### RT-PCR analysis of *AnGam* densovirus in Mos55 cells

Total RNA was extracted from Mos55 cells by TRI-reagent RT, and 10 µg RNA was subjected to DNase I and RNase A digestion for 30 min at 37°C, heat-inactivated at 65°C, and then subjected to standard phenol–chloroform:IAA extraction and isopropanol precipitation. First strand cDNA synthesis was performed using 1.0 µg untreated RNA, 0.78 µg DNase I-treated RNA, and 0.25 µg RNase A-treated RNA using the NEB Random Primer Mix and Protoscript. PCR was performed on 1 µL Mos55 cDNA in 50-µL reactions using the specified Amp1, Amp2, and *AnGam RpS7* primer pairs with Phusion DNA Polymerase. Amp1 primers: TACAAGAACAAGGCAGTTCCAGC; CCAATAAGTTATCCAATATTAGTG. Amp2 primers: TGGACTTATATCAAATTCCTATATGG; ACGGGGATCCCGGACTAATGTTGGC. *AnGam RpS7* primers: GGTGCACCTGGATAAGAACCA; CGGCCAGTCAGCTTCTTGTAC.

### Reducing redundancy in transposon family consensus sequences lists

Because most mosquito transposon annotations were derived automatically with bioinformatic prediction scripts such as the RepeatModeler package that consists of RepeatMasker, RepeatScout/TEFam, RECON, and TRF program tools (Bao and Eddy 2002; Price et al. 2005; Gelfand et al. 2007; Wheeler et al. 2013), the heuristic issue is that its efficient process generates lists of transposon families that are very redundant. Therefore, we developed different strategies for each species to mitigate overcounting of small RNAs that are elaborated in the Supplementary Text in Supplemental Materials and Supplemental Table S1.

From these consolidated lists, we applied the RepeatMasker program (Wheeler et al. 2013) to identify the genome copy numbers and genome coverages for each transposon from four organism, and we then applied small RNA counts for the benchmarking results in Supplemental Figure S1. Different merging methods were required to accommodate the different genome sizes and transposable element (TE) type compositions among the mosquito species. We treated manually curated Repbase entries as the prime standard keeping as a representative TE family consensus sequence, which was only extensive for *AnGam* and enabled quick merging just with BLAT. However, in *CuQuin*, *AeAeg*, and *AeAlbo*, Repbase entries were very few, but all other prediction entries were numerous, so for *CuQuin* and *AeAeg* we used the more specific MeShClust program to cluster TE entries and pick centroid entries we kept as representative of the merged TE family consensus sequences at the 55% similarity cutoff. But in *AeAlbo*, a nearly doubling of the number of TE species predictions, primarily from a huge expansion of LTR elements, repeatedly caused the MeShClust program to crash. Therefore, we used the less-specific CD-HIT program, also at 55% similarity cutoff, and additional repeat lengths and small RNA mapping cutoffs to reduce the redundancy in the list of *AeAlbo* TE family consensus sequences.

### Bioinformatics analysis of small RNA data sets

For these mosquito species, we adapted our bioinformatics analysis pipelines for analyzing genic/intergenic small RNA counts and analyzing transposons/virus counts (Chirn et al. 2015). Our original pipeline consisted of a series of shell, Perl, and C scripts coupled with various short read mapping packages like Bowtie as well as BLAST and BLAT (Altschul et al. 1990; Kent 2002; Langmead et al. 2009; Langmead and Salzberg 2012). Together, the pipeline determines read length distributions, assigns reads to defined lists of miRNAs and structural RNAs, such as transfer and ribosomal RNAs; it then maps remaining reads to the genome with annotation overlays that allow for binning and counting of reads mapping to genes and predicted gene models, transposon consensus sequences, and intergenic regions.

We first indexed the genome assembly file by running BWA version 1 (Li and Durbin 2010) and formatdb from NCBI. Within the genic/intergenic small RNA pipeline, small RNA reads were first trimmed by the cutadapt program (Didion et al. 2017) to remove the adaptor sequences in the 3′ end. Trimmed reads were then mapped to a collection of virus sequences using Bowtie with two mismatches (Langmead et al. 2009). Reads that were mapped to the virus were removed. Next, reads were mapped to miRNAs and structure RNAs, for example, snRNAs, tRNAs, rRNAs, and snoRNAs using Bowtie with two mismatches. Reads which were mapped to miRNAs, and structure RNAs were removed. Finally, reads were mapped to genomes using Bowtie with two mismatches to get the genic/intergenic counts using the genome GTF file. Genic counts were further categorized into 5′ UTR counts, CDS counts, and 3′ UTR counts.

The fixed step WIG file was generated by recording the normalized read counts within every window of 25 bases for the positive strand and negative strand, respectively. The wigToBigWig program was used to covert the fixed step wig file to the bigWig file which was loaded to the Broad Institute Integrative Genomics Viewer (IGV) (Robinson et al. 2011) together with the genome assembly and GTF files. Reads mapped to the intergenic regions were progressively clustered together if the normalized read count is over 0.02 within a sliding window of 25 bases. To reduce the redundancy in the genic table caused by different isoforms of a gene, the mergeBed program (Quinlan 2014) was used to consolidate different isoforms by providing the genomic location of each isoform. The isoform with the highest read counts was chosen as the representative of the gene.

Within the transposon/virus sRNA pipeline, reads were first trimmed by the cutadapt program to remove the adaptor sequences in the 3′ end. Then, trimmed reads were mapped to miRNAs with BLAST (Altschul et al. 1990). Reads that were mapped to miRNAs were removed. Then, reads were mapped to transposons using Bowtie with two mismatches and virus using Bowtie with one mismatch. Finally, the mapping patterns with respect to transposons/viruses were plotted with an R script (R Core Team 2013). Hierarchical clustering was performed by calling Python Seaborn Clustermap function using the Euclidean distance and an average linkage clustering method. Principal component analysis (PCA) was carried out by R prcomp function, with plots generated by the ggplot function. Methods for curating genic and intergenic piRNA Cluster Loci (piRCL) and predicting the piRNA targets are elaborated in the Supplemental Materials.

### piRNA ping-pong and phasing analysis

Reads were first trimmed by the cutadapt program to remove the adaptor sequences in the 3′ end. Then, trimmed reads longer than 23 nt were aligned to the genome using Bowtie with no mismatch. The genomic location and the number of times sequenced for each of the mapped reads were recorded. Using this information, we carried out autocorrelation analysis to identify periodic peaks based on a previous script from Gainetdinov et al. (2018). For 3′ to 5′ phasing analysis, autocorrelation analysis of 3′ to 5′ distance on the same genomic strands were carried out, and the *Z*-score at distance 0 was calculated, and a significant *Z*-score > 2 was observed in most cases. For 5′ to 5′ phasing analysis, autocorrelation analysis of 5′ to 5′ distance on the same genomic strands were carried out, and periodic peaks were observed on the autocorrelation scores. For piRNA ping-pong analysis, autocorrelation analysis of 5′ to 5′ distance on the opposite genomic strands were carried out and *Z*-score at distance 10 was calculated, noting *Z*-scores > 2 as significant. The siRNA duplex analysis was similar except that *Z*-score at distance 21 was calculated.

## Data access

All new deep-sequencing data generated in this study have been submitted to the NCBI Gene Expression Omnibus (GEO; http://www.ncbi.nlm.nih.gov/geo/) under accession number GSE146545. Additional curated outputs and source file details can be found at https://laulab.bu.edu/msrg/. Computational scripts are available at GitHub (https://github.com/laulabbumc/MosquitoSmallRNA) and as Supplemental Code.

## Competing interest statement

O.S.A. is a founder of Agragene, Inc., with equity interest. The terms of this arrangement have been reviewed and approved by the University of California, San Diego in accordance with its conflict of interest policies.
